# Multiple epidural steroid injections and body mass index linked with occurrence of epidural lipomatosis: a case series

**DOI:** 10.1186/1471-2253-14-70

**Published:** 2014-08-15

**Authors:** Rafael Jaimes, Angelo G Rocco

**Affiliations:** 1 The George Washington University, 2300 I St NW Ross Hall Room 456, Washington D.C 20037, USA; 2 Department of Orthopedics, Harvard Vanguard Medical Associates, 133 Brookline Ave, Boston, MA 02215, USA

**Keywords:** Epidural lipomatosis, Epidural steroid injection, Obesity, Cortisol, Sciatica

## Abstract

**Background:**

Epidural lipomatosis (EL) is an increase of adipose tissue, normally occurring in the epidural space, sufficient to distort the thecal sac and compress neural elements. There is a lack of knowledge of risk factors, impact on patient’s symptoms, and its possible association with epidural steroid injections.

**Methods:**

History, physical examination, patient chart, and MRI were analyzed from 856 outpatients referred for epidural steroid injections. Seventy patients with signs of EL on MRI comprised the study group. Thirty-four randomly selected patients comprised the control group. The severity of EL was determined by the MRI assessment. The impact of EL was determined by the patient’s history and physical examination. Logistic regression was used to correlate the probability of developing EL with BMI and epidural steroid injections.

**Results:**

EL was centered at L5 and S1 segments. The average BMI for patients with EL was significantly greater than that of control group (36.0 ± 0.9 vs. 29.2 ± 0.9, p <0.01). The probability of developing EL with increasing BMI was linear up to the BMI of 35 after which it plateaued. Triglycerides were significantly higher for the EL group as compared to controls (250 ± 30 vs. 186 ± 21 mg/dL p < 0.01). The odds of having EL were 60% after two epidural steroid injections, 90% after three epidural steroid injections and approached 100% with further injections, independent of BMI. Other risk factors considered included alcohol abuse, use of protease inhibitors, levels of stress, hypothyroidism and genetic predisposition. However there were insufficient quantities to determine statistical significance with a degree of confidence. The impact of EL on patient’s symptoms correlated with EL severity with Spearman correlation coefficient of 0.73 at p < 0.01 significance level.

**Conclusions:**

The BMI and triglycerides levels were found to be significantly elevated for the EL group, pointing to an increased risk of EL occurrence in progressively more obese US population. The data also revealed a strong correlation between the number of subsequent epidural steroid injections and EL occurrence calling for caution with the use of corticosteroids.

## Background

Epidural lipomatosis (EL) is an increase of adipose tissue, normally occurring in the epidural space, sufficient to distort the thecal sac and even compress neural elements. EL was first reported in 1975 with the use of corticosteroids to prevent rejection of a kidney transplant [1]. A 2005 review of the 104 cases of EL in the literature identified four categories associated with EL: i) exogenous steroid use, ii) obesity, iii) endogenous steroid excess and iv) idiopathic [2]. A 2008 review of the world literature found 111 cases of EL with 56% secondary to corticosteroids*,* exogenous or endogenous [3]. Thoracic EL was associated with corticosteroid use and lumbar EL with obesity [3]. Tissue surgically removed from patients with EL showed histologically normal, un-encapsulated fat in increased amounts [2-7]. Although the existing literature clearly points to a link between obesity and steroid administration, there has not been a systematic assessment of risk factors that can lead to EL, particularly the effect of multiple steroid injections for lower back pain. We sought to address this question using an analysis of 856 patients referred to our clinic for lower back pain.

## Methods

**Figure 1:**
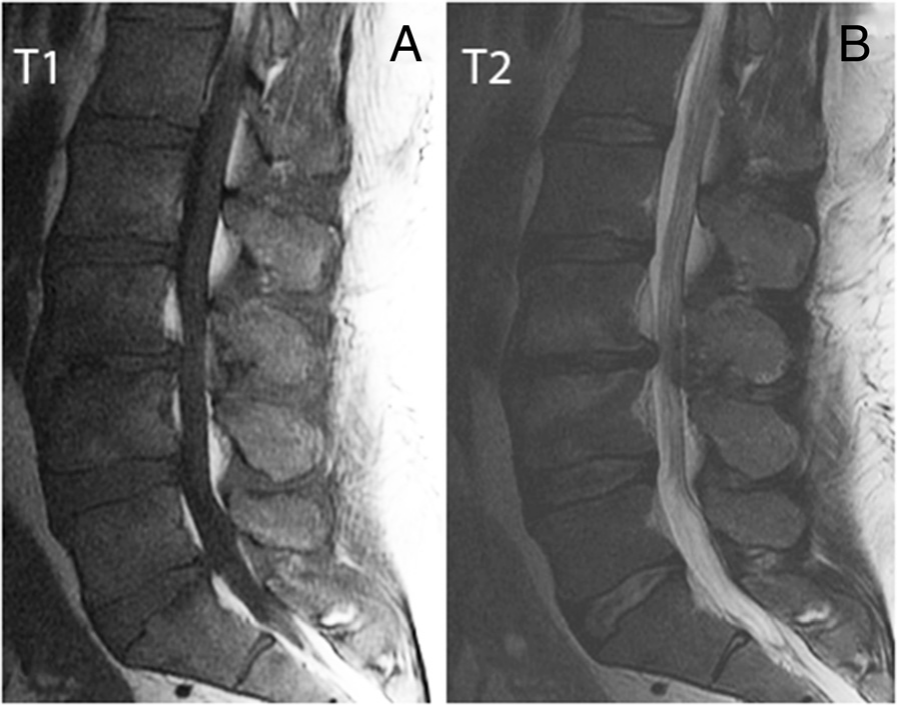
**Comparison of the sacral area on T1 and T2 sagittal images. (A)** The T1 on the left shows the thecal sac is partially obscured by EL. **(B)** The T2 sagittal MR image on the right shows a clear outline of the thecal sac ending at mid S2.

**Figure 2:**
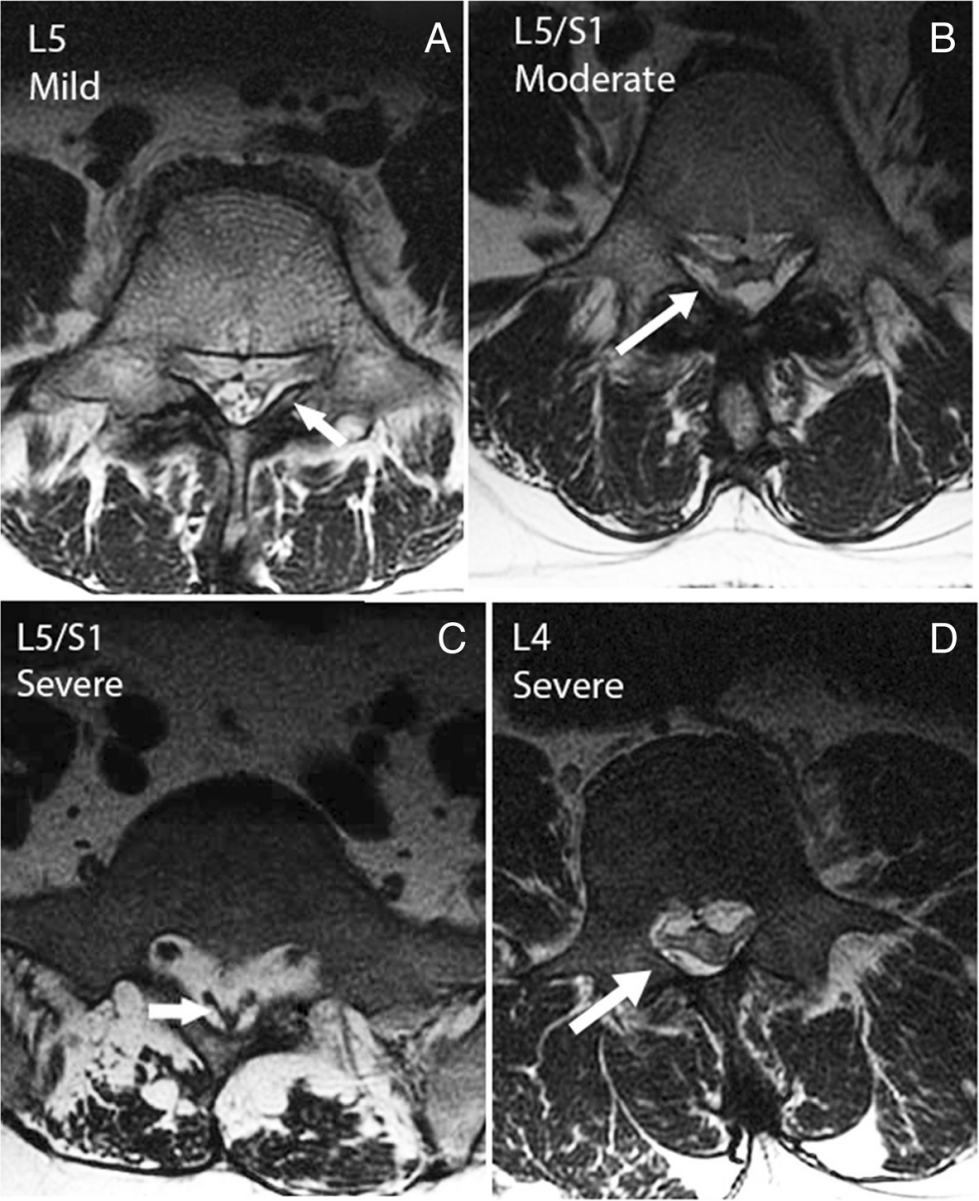
**Severity of EL can be determined from physical features. (A)** Axial image through the body of L5 with early EL. The L5 nerve roots are visible from thecal sac to Lateral Recesses. Epidural Lipomatosis (EL) enhances the visibility of the L5 roots. The arrow points to the indentation of the thecal sac. **(B)** Epidural fat tethering dural sac producing stellate appearance and moderate EL. **(C)** Axial MR image through the body of S1 with severe EL. The arrow points to the thecal sac in the shape of a Y (pathognomonic for severe EL). The buttock muscles are infiltrated with fat and the overlying subcutaneous tissue is excessive. **(D)** Axial MR image through the body of L4 with EL, compressing the roots and thecal sac. Since the neural elements normally occupy a larger area of the spinal canal at L4 than L5/S1, the compression by EL is considered severe.

IRB approval to collect data from the records was obtained at Harvard Vanguard Medical Associates (HSC# 6.6.08, Coeus# 0809000134). ESI was performed in the appropriate interspace using previously described protocol [8] with a standard mixture of 120 mg depomedrol in 3 mL normal saline, both preservative free.Review of hard copies of MR images available during the clinical examination and assessment revealed that 52 of 856 patients had EL on the basis of deformation of the thecal sac and/or the nerve sheath by lipoid on the T1 films (Figures 1 and 2A). Data was analyzed in a nonclinical setting after clinical treatment ended. To generate a subpopulation of internal controls without EL, drawn from the same clinical cohort, a convenience sample based upon serial selection of one case from each dozen seen serially was employed. Eighteen of these 52 patients from the remaining 804, contingent on the availability of a complete record, were found to have mild EL. These eighteen patients were combined with the original 52 patients to form patients with EL (70 patients). The remaining 34 patients with no signs of EL formed the non-EL control group. A large number of EL patients vs. control group allowed us to further subdivide the EL group into different EL severity categories. The control group with greater than 30 samples provided enough statistical power to show significant comparisons between groups.

**Figure 3:**
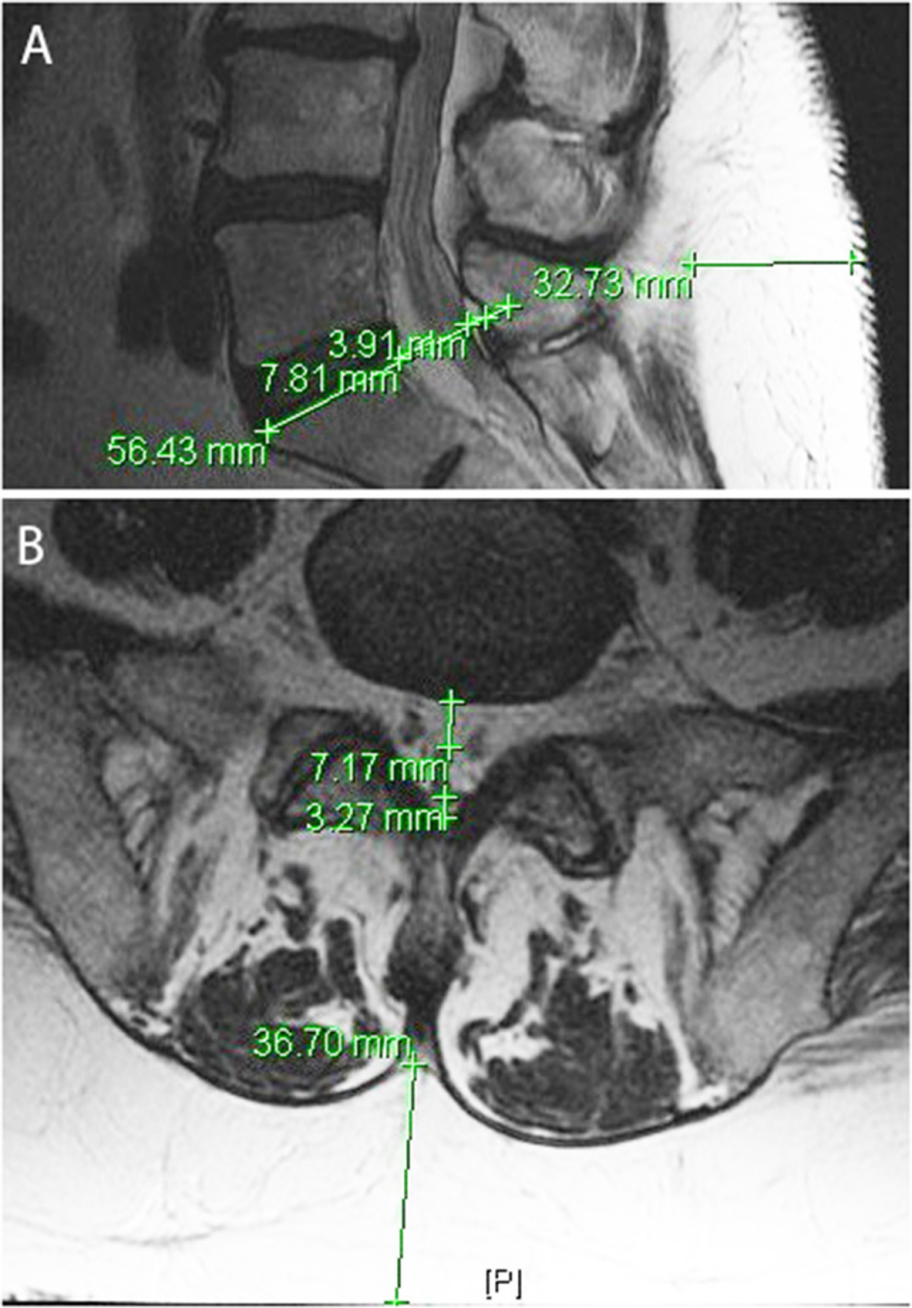
**Comparison of sagittal and axial images. (A)** Sagittal T1 showing epidural fat measurements along a line tangent to the superior cortex of S1, anterior (7.8 mm) and posterior (3.9 mm). The thickness of posterior subcutaneous fat was measured horizontal to the L4/L5 interspace, line labeled 32.7 mm. **(B)** Axial image showing measurement discrepancy: anterior epidural fat is 7.2 mm, posterior epidural fat is 3.3 mm, and posterior subcutaneous fat is at least 36.7 mm.

The following data were obtained from records: date and number of previous ESIs, age, sex, BMI range, presence of diabetes, HA1C, mean glucose, random glucose, triglycerides, cholesterol, LDL, HDL, CPK, SGPT, SGOT, ALK- PHOS, uric acid and creatinine.The severity of EL was graded by visually assessing the degree of compression and distortion of the thecal sac on all T1 images. The ratio of cross sectional area of epidural fat to that of the dural sac was used as a guide. In the L5 and S1 area, if epidural fat occupied a cross-sectional area up to that of the dural sac, it was considered mild (Figure 2A); up to twice was moderate (Figure 2B); and greater than twice was severe (Figure 2C). However, in the more cephalad segments, the neural elements occupy a larger section of the spinal canal thus; a smaller area of epidural fat can produce severe compression as in the upper lumbar spine (Figure 2D). The grading was done on a visual basis, simplified for use in the clinic as none, minimal, moderate and severe EL. To confirm visual grading scheme, linear measurements of epidural fat on MRI were made in both EL and non-EL control groups using an electronic ruler on Fuji PACS system: the epidural fat, anterior and posterior to the thecal sac was measured in the sagittal image tangent to the superior cortex of S1; the posterior subcutaneous fat was measured horizontal to the L4/L5 interspace (Figure 3A). The corresponding image from the axial plane showing measurement discrepancies is shown in Figure 3B.

All statistical analysis was performed in SAS 9.3. BMI, triglycerides, cholesterol, and fat from sagittal measurements (total epidural and posterior subcutaneous) were compared with Wilcoxon-Mann–Whitney (WMW) and/or T-test as noted. Statistical significance was defined at p-values of less than 0.01.

A Spearman correlation test was performed between values of BMI and the EL severity, BMI and the EL impact on patient’s symptoms, and the EL severity vs. the EL symptoms. Equality of gender was shown using a binomial exact test. The number of epidural steroid injections (ESI) or BMI vs. probability to develop EL was computed by odds ratio and logistic regression (Eq. 1).

(1) $$\log_{e}\left(\frac{p_{i}}{1-p_{i}}\right)=\beta_{0}+\left(\beta_{1}*\mathrm{Var}\right)$$

Where *β*_0_ and *β*_1_ are the solved coefficients, *Var* is the number of ESI given or BMI, and *p*_*i*_ is the probability of acquiring EL. The correlation was considered to be significant for variables with a p-value of less than 0.01. Logistic regression was calculated separately for BMI and ESI to ensure independence.

## Results

### Demographics

After above mentioned selection from an original pool of 856 patients, there were a total of 104 patients analyzed. This included 70 patients with EL and 34 without EL. There were 43 men and 61 women in the study. A binomial test of these two groups resulted in a p < 0.05, confirming that the numbers of men and women in both groups were statistically equal. The average age of the patients was 61.8 ± 2.8 years. Those with EL had an average age of 61.7 and those without, 61.8.

### Identification of EL

**Figure 4:**
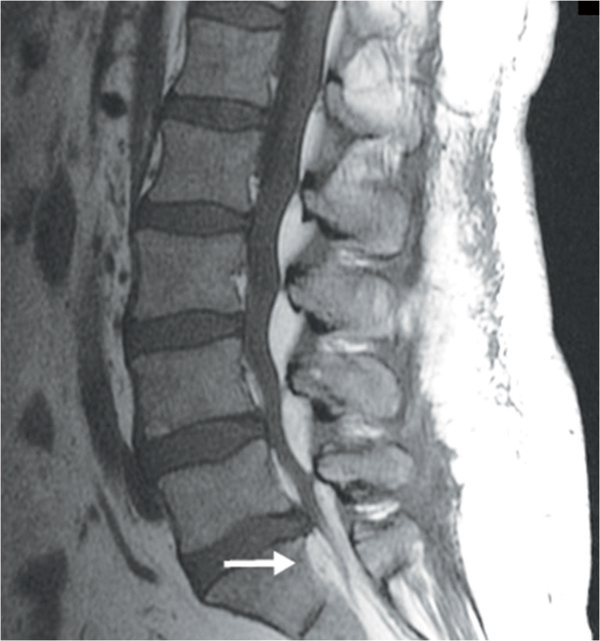
**T1 sagittal image confusable with arachnoiditis.** The arrow at S1 points to excessive anterior fat and a compressed thecal sac.

T1 weighted MR images were used for identification of EL as displayed in the sacral area on the sagittal image (Figure 1A). As shown in Figure 1B, T2 weighted images may not allow enough contrast between fluid and fat.Out of 70 EL patients, 46 were classified as mild. Figure 2A exemplifies mild EL: enhanced visibility of the L5 roots and mildly indented thecal sac. Sixteen patients were classified with moderate severity, as exemplified in Figure 2B: enhanced L5 roots and cross sectional area of epidural fat greater than that of the thecal sac. Figure 2C is an image at L5/S1 and is indicative of severe EL: the thecal sac is compressed to form a Y-shape. There were eight patients with the classification of severe EL. Figure 2D is an image taken at the L4 level and illustrates the case where although the thecal sac and epidural fat are equal, the neural elements are compressed significantly thus considered severe.A misdiagnosis that may occur is that arachnoiditis is confused with EL. The sagittal MR image may show severe EL and an irregularly compressed dural sac confusable with arachnoiditis (Figure 4). Using the T1 image, enhanced contrast will help the clinician to distinguish the two.

### Linear image measurements of epidural and of subcutaneous fat

**Figure 5:**
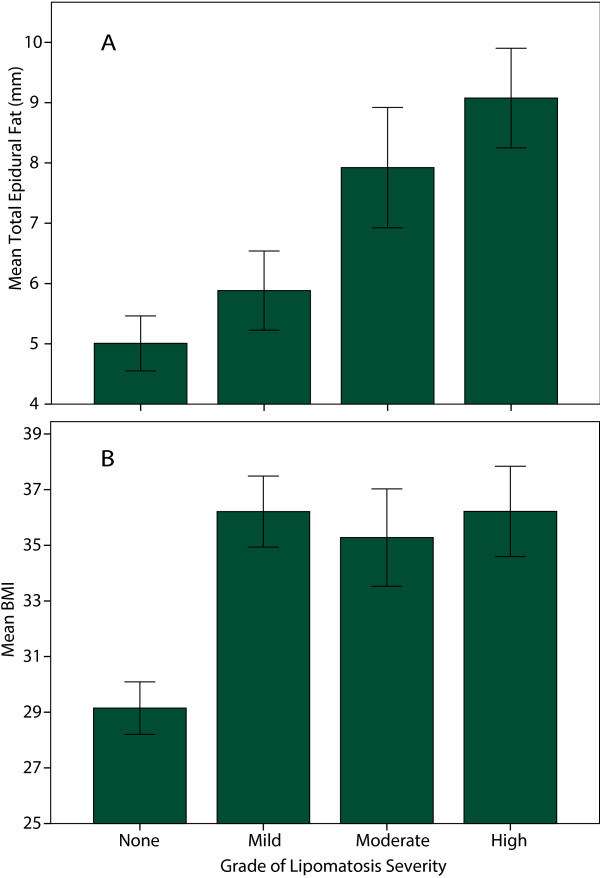
**Grade of lipomatosis severity increases with epidural fat and BMI. (A)** Holistic grading approach of EL scales with quantitative total epidural fat measurements. **(B)** BMI was statistically different between control and EL group, but not different between grades of EL.

The anterior and posterior epidural, and posterior subcutaneous fat measurements were made in both EL and non-EL control groups using a Fuji PACS system in the sagittal (Figure 3A) and axial planes (Figure 3B). The measurements of fat varied slightly between the orientations. Measurements in the sagittal plane were more consistently replicable than in the axial plane. Sagittal epidural fat measurements were combined and reported as total epidural fat. The total epidural fat for patients with EL was significantly greater than the total for non-EL control group as determined by WMW test (7.2 ± 0.4 mm vs. 5.0 ± 0.4 mm, p < 0.01). Qualitative visual analysis of lipomatosis grades revealed a nearly linear correlation with total epidural fat measurements (Figure 5A). The comparison of total epidural fat of males and females showed no difference within their respective groups. However, females had significantly more posterior subcutaneous fat than males within their respective groups, p < 0.01. Posterior subcutaneous fat was also significantly greater in patients with EL than non-EL (37.5 ± 2.6 mm vs. 27.9 ± 2.0 mm, p < 0.01).

### Increased body mass index and triglycerides are associated with epidural lipomatosis (EL)

**Figure 6:**
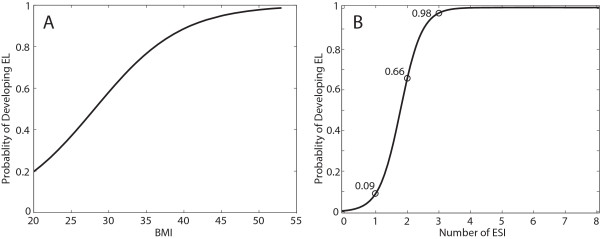
**Probabilities of developing EL computed by logistic regression. (A)** Probability computed with BMI as the variable. **(B)** Probability computed with number of prior epidural steroid injections as the variable.

The average BMI for all patients in the study was 33.7 ± 0.7 (obese). The average BMI for men was 33.4 ± 1.1 and 33.6 ± 0.9 for women. Both T-test and WMW test had p-values of 0.8 and 0.15, respectively, suggesting no difference in BMI based on gender.The average BMI for patients with no EL was 29.2 ± 0.9 and those with EL was 36.0 ± 0.9. A weighted T-test and WMW test had p < 0.01, suggesting there is a difference in BMI between the two groups. An ANOVA with Tukey post-hoc test on BMI between different grades of lipomatosis revealed significant differences for all grades of EL severity when compared to the control group (p < 0.01), but no difference comparing the different grades of lipomatosis (Figure 5B). BMI was found to be statistically significant for the logistic regression model with a p < 0.01. The probability of developing EL was linear with increasing likelihood as BMI increases, until a BMI of 35 after which point it plateaus (Figure 6A).

Those with EL had an average triglycerides value of 250 ± 30 and those without EL had an average of 186 ± 21 mg/dL. To account for the fact that triglycerides values were positively skewed in both groups, a WMW test was used. Triglycerides in the EL group were significantly elevated compared to the control group with p < 0.01. Triglycerides do not independently determine the probability of developing EL with a p > 0.01 in a logistic regression model.

### Severity of lipomatosis on patient’s symptoms

The chief complaints, causes, and associated complaints were determined from history and physical examination as specified in Table 1. Twenty-eight (40%) EL patients complained of pain on walking relieved in a few minutes with sitting (neurogenic claudication). Forty-two (60%) complained of sciatica. EL was the primary cause of sciatica in 9 of 42 (21%) but may be contributory in other patients. The impact of EL on patient’s symptom impact was then graded as none, minimal, moderate and high. The Spearman chi-square p-value for EL severity vs. impact was <0.0001 showing that there is strong association between EL severity and symptomatic impact (Table 2). The early stages of the development of EL had low or no symptomatic impact.

**Table 1:** Categories of Chief Complaint

**Chief complaint**	**Primary causes**	**EL group (n = 70)**	**Associated complaints**
Neurogenic Claudication (n=28)	Triad^1^	14	No Sciatica
	Foraminal Stenosis	9	Sciatica
	DJD^2^	5	Facetal Pain
Sciatica (n=42)	HNP^3^	25	Radicular pain, sensory and/or motor deficit
	EL^4^	9	
	Foraminal Stenosis	8	

^1^Disc bulge, thickened ligamentum flavum, facet hypertrophy (spinal stenosis). ^2^Degenerative joint disease (spondylolisthesis and/or facet arthropathy). ^3^Herniated nucleus pulposis. ^4^Epidural lipomatosis.

**Table 2:** Severity of EL and its relationship to gradation of impact

**Impact**	**None**	**Minimal**	**Moderate**	**Severe**
**EL severity**				
**Mild**	32	13	1	0
**Moderate**	0	3	11	2
**High**	0	2	3	3

The Spearman correlation severity vs. impact (R^2^ = 0.73, p < 0.0001).

### Epidural steroid injections, stress, & alcohol consumption

The number of ESI deliveries was significant in an independent logistic regression model with a p < 0.01. Absence of ESI deliveries or one ESI delivery did not increase the patient’s odds of developing EL. After two ESI the odds of developing EL was 66%. After three ESI, the odds were 98%. Four or more ESI increased the odds approaching 100%.

The average number of ESI delivered in the patients with no EL was 1.0 ± 0.0, and there were no patients who received more than one. The average number of ESI delivered was 1.8 ± 1.5 to the EL group. There were two patients in the EL group who did not receive any ESI. Their BMI were 40 and 42.

It was noted in the charts of ten patients in the EL group who self-reported stress and eight who self-reported alcohol consumption. No patients in the control group reported stress or alcohol consumption.

## Discussion

### Definition, location, severity, impact

The definition of EL presently available, “an excessive accumulation of normally occurring fat in the epidural space” [2-7,9-11], does not provide a definitive boundary between the normal and excessive amount of fat in the epidural space thus we added *visible deformation of the thecal sac/neural elements* to provide a more definitive end point.

**Figure 7:**
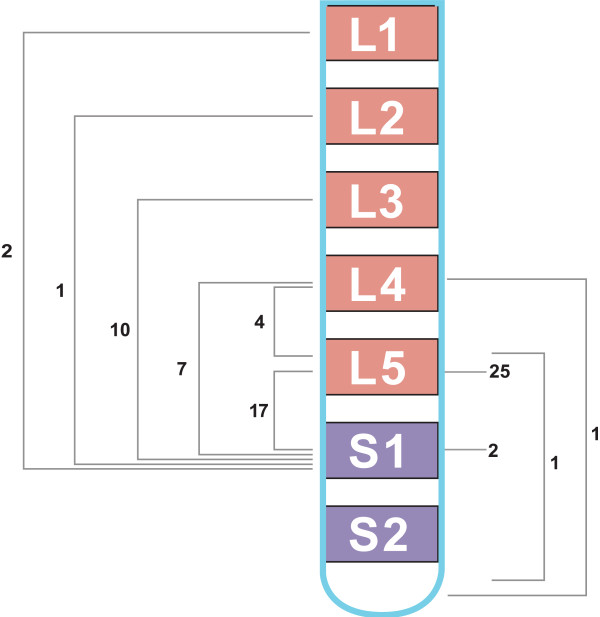
**Location and extent of involvement of EL.** The brackets show the extent of EL and labeled locations show the respective number of cases involved.

Our observation that EL is centered on L5 to S1 extending to include some cephalad and caudad segments, Figure 7, is consistent with the findings of Borre et al. [4].

Severity was correlated with impact. Pinkhardt et al., found a “very insecure association (of EL) with clinical symptoms” [11]. They made measurements in L5/S1 axial images according to Borre [4] retrospectively, excluding patients with deformities whereas our study included all sagittal and axial T1 MR images. The MRI findings must be put in the context of the history and physical exam.The mean BMI for the control group was under 30, but approximately 34 for EL patients indicating that there is step response beyond the threshold of obesity (Figure 5B). With a BMI of 28, the probability of EL is only 50% which hints there are other contributing factors. Other causative factors, including, but not limited to ESI and elevated triglycerides, will significantly contribute to the probability of developing EL up to a BMI of 35. The probability of developing EL is linear with increasing BMI until a value of 35, at which point the probability curve begins to plateau (Figure 6A). Once BMI is greater than 35, the other causative factors have less of a contribution toward the probability of developing EL, but may contribute to the grade of EL severity. The grade of EL severity is not correlated with BMI (Figure 5B).

### The causes of epidural lipomatosis

The probability of developing EL is linear with increasing BMI between 28 and 35. BMI of 28 acts as a threshold for developing EL and at a BMI 35 its influence begins to wane. There are other factors involved which will be discussed later. Obesity, BMI over 30, is an imputed cause of EL [2-4,10,12,13] which provides a milieu for the enzyme, 11β-HSD-1 to convert corticosteroids to cortisol [14], Diet induced obesity leads to an increase in 11β-HSD-1 which increases cortisol by the local conversion of corticosteroids to the biologically active cortisol [14]. Corticosteroids also, an imputed cause of EL [2-4,6,15-19], fit into the schema by providing the substrate for the formation of cortisol.

Study of the physiology of cellular liporegulation in rodents sheds some light on the role of Leptin in human metabolism of fat [14]. Leptin is required for normal liporegulation in tissues just as insulin is required for normal glucoregulation. In over-nutrition, leptin allows extra fat to be deposited in body fat without metabolic injury to non-adipose tissues. An increase in cortisol leads to leptin resistance; exacerbated by glucocorticoid administration [20] and ameliorated by adrenalectomy [21]. If leptin is overwhelmed, then deposition of fat may take place in the liver, heart, muscles and pancreas causing lipotoxicity or lipoapoptosis [14]. The liver has a margin of safety by exporting triglycerides in the form of very low density lipoproteins (VLDL); the muscle can metabolize triglycerides as a result of exercise, but the pancreas has neither of these options [14].

A phase IIB study in patients with Type 2 diabetes given an antagonist to the enzyme 11β-HSD-1 responsible for conversion of corticosteroids to cortisol showed increased insulin sensitivity and improvement in: glycemic control, total cholesterol, LDL-cholesterol and triglycerides [22]. Studies are needed to show if 11β-HSD-1 and cortisol lead to EL.

Hypertriglyceridemia may develop due to overeating and/or under-exercising [23,24]. Obesogens can alter regulation of energy balance to favor weight gain and obesity [25]. Also, an increase in adipose tissue cortisol can lead to lipolysis with increased triglycerides [26].

### EL and ESI

Reports have related the development of EL to the administration of epidural steroid injections [27-30]. Four patients were refused ESI because the EL was severe and structural defects were minimal. Although the probability of developing EL is over 90% after four ESI, the severity and thus symptomatic impact of EL may not be significant. The incidence of EL was not assessed in this study. The corticosteroid from the ESI provides the substrate for the formation of cortisol.

The response to stress, as formulated by Selye [31], causes the release of corticotrophin by the pituitary; cortisol, epinephrine and other hormones by the adrenal gland. The cortisol, thus produced, augments the cortisol from the adipose tissue, ultimately promoting the metabolic syndrome [14,15,20,32-34]. The case-controlled study by Brunner et al. provides evidence that chronic stress may be a cause of metabolic syndrome [35]. Only our patients that developed EL reported stress.

Studies of alcohol abuse on metabolic syndrome are cogent since EL and metabolic syndrome share many components. Some studies have shown a beneficial effect of alcohol on the development of metabolic syndrome. However Baik found an increased risk of metabolic syndrome was associated with “obese, heavy liquor drinking persons” [36]. Alcohol feeding of patients on a metabolic ward increased plasma triglycerides, the obese responding with a 45% increase in the production of VLDL-TG [37]. Thus, the caveat: mild or moderate alcohol consumption may be protective, but only in persons who are not obese. Only patients in the EL group reported alcohol consumption.

## Conclusion

The development of EL is multi-factorial, the risk factors being intertwined and additive. Obesity is the principal risk factor associated with lumbar epidural lipomatosis. Lumbar EL may worsen after the first ESI injection. The symptomatic impact of EL is correlated with its severity. EL may be the principal cause of sciatica and secondary cause of neurogenic claudication. The present trend in obesity portends a marked increase in epidural lipomatosis [38]. Weight loss programs have reversed EL and its symptoms, including neurogenic claudication and cauda equina compression [39-43]. The density of cases is sufficient to formulate controlled studies.

## Competing interests

The authors declare they have no competing interests.

## Authors' contributions

AGR is the anesthesiologist who treated all 856 patients, reviewed all MRIs for EL, and graded EL on the visual scale. AGR conceived the study and drafted the original manuscript. RJ carried out all statistical tests. Both authors made linear measurements to the MRIs and revised the manuscript. Both authors read and approved the final manuscript.

## Pre-publication history

The pre-publication history for this paper can be accessed here:

http://www.biomedcentral.com/1471-2253/14/70/prepub
